# Person-Centered Assessment of Behavior Changes in People With Dementia

**DOI:** 10.1093/geroni/igab046.1011

**Published:** 2021-12-17

**Authors:** Emilee Ertle, Benjamin Mast, Gail Mountain, Ann Kolanowski, Esme Moniz-Cook, Margareta Halek

**Affiliations:** 1 University of Louisville, Louisville, Kentucky, United States; 2 University of louisville, louisville, Kentucky, United States; 3 University of Bradford, West Yorkshire, England, United Kingdom; 4 Penn State, University Park, Pennsylvania, United States; 5 University of Hull, Hull, England, United Kingdom; 6 Witten/Herdecke University, Witten, Nordrhein-Westfalen, Germany

## Abstract

Behavioral and psychological symptoms of dementia are increasingly being reconceptualized as expressions of distress and unmet needs. Measures that evaluate context are needed to increase our understanding of factors that influence these expressions. This review evaluated measures for two common behavioral states that are experienced as challenging for caregivers: apathy and resistance to care. A systematic literature search identified measures of apathy or resistance to care for people living with dementia. Eight measures of apathy and three measures of resistance to care were identified. Reliability and validity of these measures were evaluated using the COSMIN framework, as well as reported contextual factors within which the behavior occurs. The identified measures had fair to good reliability and validity in people living with dementia. However, available measures need to move beyond symptomatic constructs for this complex paradigm, and toward the varied interpersonal and contextual factors associated with behavioral expression.

